# Development of the Nurses’ Attitudes Toward Violence by Patients and Patients’ Relatives Scale: A Methodological Study

**DOI:** 10.3390/bs16091665

**Published:** 2026-09-16

**Authors:** Deniz Gür, Ülkü Baykal

**Affiliations:** 1 Vocational School, Atlas University, 34403 Istanbul, Türkiye; 2 Department of Nursing Management, Florence Nightingale Faculty of Nursing, Istanbul University-Cerrahpasa, 34320 Istanbul, Türkiye; ulku.baykal@iuc.edu.tr

**Keywords:** workplace violence, nurses’ attitudes, patient violence, psychometric properties, scale development

## Abstract

Violence against nurses is a global occupational health and safety issue, yet instruments that assess nurses’ attitudes towards violence from patients and their relatives are limited. This methodological study involved the development and evaluation of the Nurses’ Attitudes Towards Violence by Patients and Patients’ Relatives Scale. In the qualitative phase, 39 nurses were interviewed in depth until data saturation was reached, generating a pool of 61 items. Content validity with 15 experts (CVI = 0.90) yielded a draft of 57 items. The original quantitative sample (*N* = 608) was randomly divided into exploratory (*n* = 304) and confirmatory (*n* = 304) subsamples. Principal axis factoring with ProMax rotation supported a 36-item, three-factor solution that explained 52.3% of the common variance. This solution comprised the following factors: perception and causes of violence (20 items); effects of violence (13 items); and response to violence (3 items). In the original confirmatory subsample, the final model with six theory-based residual covariances was subjected to WLSMV confirmatory factor analysis, showing an overall acceptable fit (χ^2^(585) = 1297.5, χ^2^/df = 2.22, CFI = 0.900, TLI = 0.892, RMSEA = 0.063 [90% CI 0.059–0.068], SRMR = 0.093). A robustness wave involving 45 additional nurses, conducted between 27 August and 2 September 2026, produced almost identical results when combined with the confirmatory subsample (*n* = 349). Supplementary analyses supported composite reliability and discriminant validity, and found that measurement invariance was supported across institution type, whereas findings regarding sex invariance were interpreted as preliminary. In the original sample of *N* = 608, Cronbach’s α was 0.943, 0.930 and 0.842 for the three factors, and 0.950 for the total scale. Two-week test–retest reliability was r = 0.976. The scale provides a multidimensional measure of nurses’ evaluative-attributional beliefs, perceived effects and responses to violence from patients and relatives.

## 1. Introduction

Violence against healthcare workers is widespread and constitutes a significant occupational safety issue (Fiorino et al., 2026; Mahmoudi et al., 2026). It attracts considerable attention because it has an adverse effect on healthcare workers (Dopelt et al., 2022). Furthermore, violent incidents disrupt care processes, hindering the effective delivery of healthcare services (Seddik et al., 2023).

Violence is a multidimensional concept that can be interpreted in different ways in legal, clinical, public health and experiential contexts. While legal definitions depend on whether particular behaviours meet established legal thresholds, lived experience encompasses a broader range of behaviours that are perceived as hostile, coercive, harmful or intimidating. In this study, violence perpetrated by patients and their relatives was defined in accordance with the framework developed by the International Labour Organization (ILO), the International Council of Nurses (ICN), the World Health Organization (WHO) and Public Services International (PSI) to address workplace violence in the healthcare sector. Accordingly, violence encompasses both physical and psychological forms, including verbal abuse and threats, as perceived and reported by nurses. This operational definition recognises that nurses’ experiences and interpretations of violence may not always align directly with formal or legal definitions, and therefore these definitions should be taken into account when interpreting the findings.

According to meta-analysis data, 62% of healthcare workers encountered patient or visitor violence in the previous year. According to a WHO report (2020), between 8% and 38% of healthcare workers experience physical violence at some point in their careers. The number of healthcare workers subjected to verbal assault or threats is even higher than these figures suggest (Liu et al., 2019; World Health Organization, 2022). The prevalence of violence at this level highlights the need for a comprehensive approach to addressing its impacts, causes and prevention.

Nurses, numbering approximately 28 million and forming the majority of the entire healthcare workforce (World Health Organization, 2020), are at twice the risk of being subjected to violence and five times the risk of experiencing physical violence compared to physicians (Berger et al., 2024). Nevertheless, violence, most often perpetrated by patients and their relatives, is frequently perceived as a routine part of work in clinical settings (Alfuqaha et al., 2022; Berger et al., 2024; Connell et al., 2026). The normalisation of violence perpetrated by patients and visitors, coupled with a lack of knowledge about how to report it, fear of blame, a lack of support, feelings of hopelessness and the complexity of the reporting process, all hinder reporting of violence (Dunsford, 2022; Spencer et al., 2023; Elsharkawy et al., 2025; Lee et al., 2025). As more than 80% of violent incidents go unreported, access to detailed information about violence against nurses is limited (Christensen & Wilson, 2022; Mahmoudi et al., 2026).

According to research, the emergence of violence is influenced by various factors, including patient-related variables such as the severity of the illness, substance abuse or alcohol use (Pol et al., 2019; Perkins et al., 2020; Timmins & Timmins, 2021), psychiatric disorders (Salzmann-Erikson & Yifter, 2020), psychological issues and a history of violence (Cho et al., 2023). Employee youth or inexperience, as well as a lack of communication skills, are also contributing factors in cases of violence (Bagnasco et al., 2024; Yang et al., 2025). Environmental factors, particularly in emergency departments, such as long waiting times, continuous 24/7 services and high patient volumes, which put pressure on staff, can also trigger incivility (Wirth et al., 2021; Xie et al., 2025). Furthermore, systemic issues related to the healthcare system may cause such violence. The lack of legislation against violence in healthcare, the commercialisation of health services and inadequate access to healthcare can provoke tensions between patients and employees (Wu et al., 2022).

The ILO, ICN, WHO, and PSI have long emphasised the need for policies to prevent and address violence against healthcare workers (International Labour Office et al., 2002). Existing efforts have focused predominantly on managing violence rather than reducing it (Spelten et al., 2020; Timmins & Timmins, 2021).

For affected employees, violence can have long-term consequences such as sleep disturbances, stress, an increased desire to leave their job and burnout (Dunsford, 2022), as well as minor to severe physical injuries (Cho et al., 2023). Violence towards nurses also impacts caregiving processes. Following exposure to violence, nurses continue to provide patient care despite ethical concerns (Dunsford, 2022). They may then face the dilemma of either stopping or delaying care services in order to consider the patient’s needs (Aljohani et al., 2021; Hou et al., 2022), or of balancing personal safety with providing patient care (Beattie et al., 2020).

The international literature indicates that instruments designed to measure workplace violence primarily assess the frequency and types of violence experienced by healthcare workers, the reasons why such violence is not reported through legal channels, and the strategies employed to prevent it. Various instruments focusing on attitudes have been developed to evaluate these dimensions of the learning environment. One such instrument is the Factors Affecting Patient Aggression Scale (FAPAS), which was created by Lepiešová et al. (2021) to evaluate nurses’ perceptions of the factors that increase the risk of patient aggression. It is structured into six subscales: gender aspects; physical and emotional distress; nurse-related factors; nurse shift organisation factors; patient-related factors; and nursing environment factors. This scale addresses the antecedents of aggression rather than the nurses’ attitudinal responses.

The Hospital Violence Attitude Scale-18 (HVAS-18), developed by Ha et al. (2015), comprises 18 items distributed across five factors: awareness; response; reaction; and consequences for nursing and the perpetrator. However, it lacks an integrated framework for causal evaluation and behavioural responses. Similarly, the Perception of Aggression Scale (POAS), introduced by Jansen et al. (1997) and later re-evaluated for psychometric properties by Nam et al. (2022), focuses on nurses’ cognitive evaluations of aggression, but does not address its determinants or the strategies nurses use in response. Together, these instruments show that current attitude scales tend to focus on one dimension rather than providing a comprehensive tool that assesses violence, its perceived causes, and nurses’ behavioural or coping responses. This is evident in the FAPAS’s focus on perceived causes of aggression, the HVAS-18’s multifactorial yet response-centred structure, and the POAS’s exclusive cognitive evaluation approach.

Currently, there is no comprehensive scale that simultaneously assesses nurses’ attitudes towards violence by evaluating violence, identifying perceived causes and assessing the intervention or coping strategies employed. The scale developed in this study aims to address this issue by providing nurses with a multidimensional assessment tool for violence committed by patients and their relatives. By combining these three dimensions, this scale is anticipated to meet the measurement needs identified in the literature more effectively than existing instruments.

This study aimed to develop a measurement tool to assess nurses’ attitudes toward patient and patient-related violence. This study is based on the doctoral dissertation titled “Development of the Attitude Scale for Nurses Toward Patient and Patient Relative Violence,” completed in 2023 (Gür, 2023).

## 2. Materials and Methods

### 2.1. Design

**Figure 1:**
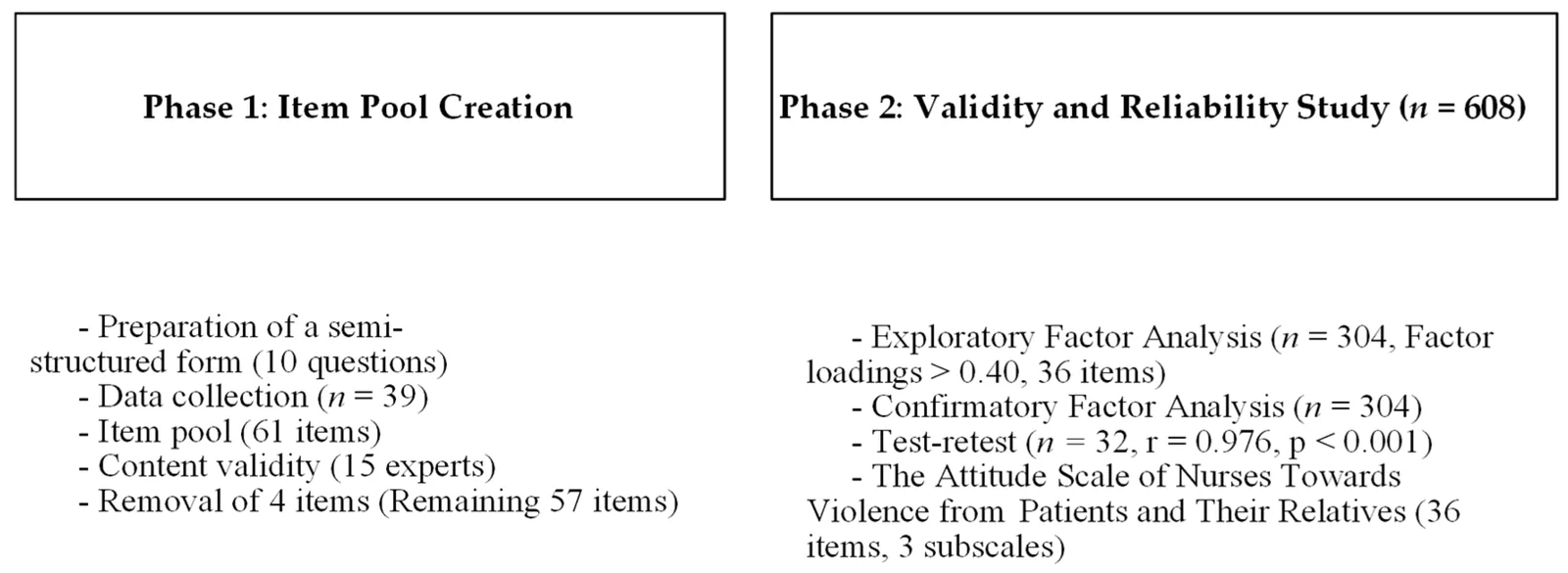
Phases in Developing the Attitude Scale for Nurses Toward Patient and Patient Relative Violence.

This methodological study comprised two phases. In the first phase, a qualitative method was used to create an item pool. The second phase involved evaluating the validity and reliability of the developed measurement tool (Figure 1).

### 2.2. Phase 1: Creation of the Item Pool

#### 2.2.1. Data Collection

This study applied a qualitative research method, which is frequently used in scale development studies and enables the collection of rich data (Neuman, 2014; Creswell, 2013/2016). Individual in-depth interviews were conducted with 39 nurses. After interviewing the 39th participant, it was evident that responses were becoming repetitive and no new substantive codes or themes were emerging. Consequently, data saturation was deemed to have been reached and the interviews were concluded.

Following the interviews, the written reports were systematically organised through coding and thematisation. Four main themes were identified: ‘Definition of violence’, ‘Violence in healthcare’, ‘Violence against nurses’, and ‘Prevention of violence in healthcare’. Thirteen sub-themes and 38 codes were then created from the texts under these themes (Gür, 2023).

Data were collected from the sample of hospitals using the snowball sampling method. Initial contact was made with intermediary nurses to identify participants who had experienced violence. After interviewing one nurse, individuals recommended by that nurse were contacted (Creswell, 2013/2021). The sample group was formed to ensure maximum diversity of the data. Interviews were scheduled with nurses who agreed to participate in the study. This approach is valued in qualitative research as it reduces the risk of selection bias (Malone et al., 2014). A semi-structured interview form containing 10 questions, which was prepared by the researcher with the help of literature, was used. The researcher recorded the interviews using a Sony digital voice recorder (Sony Corporation, Tokyo, Japan) and transcribed them verbatim. The audio recordings were transcribed verbatim on a computer.

#### 2.2.2. Qualitative Analysis and Content Validity

The transcripts were analysed using Colaizzi’s (1978) inductive content analysis method. The recordings were transcribed verbatim and significant statements related to the phenomenon were identified. These statements were then coded and organised under sub-themes and themes. The fit between these themes and the original accounts was then checked and the fundamental structure of the phenomenon was described.

Two researchers coded the transcripts independently. First, each researcher reviewed and coded the transcripts separately. They then compared the resulting code lists, discussing any differences in wording, grouping or interpretation until consensus was reached. The two analyses showed considerable conceptual overlap, and the final codebook, sub-themes and themes were agreed upon jointly. No formal inter-coder reliability coefficient (e.g., Cohen’s kappa) was calculated; agreement was established through a consensus-based coding process, which is consistent with qualitative approaches that use negotiated agreement (O’Connor & Joffe, 2020).

Four main themes (‘definition of violence’, ‘violence in healthcare’, ‘violence against nurses’, and ‘prevention of violence in healthcare’), 13 sub-themes, and 38 codes were obtained (Gür, 2023). The items were written based on the participants’ own statements and were categorised under four content domains: perception of violence (10 items), causes of violence (20 items), responses to violence (14 items) and effects of violence (17 items), totalling 61 items.

The Davis Technique was employed to evaluate the content validity ratio (CVR) of the items. Opinions were obtained from fifteen experts in management, psychiatry, the Turkish language and scale evaluation. The experts rated each item on a scale from “appropriate” (4) to “not appropriate” (1) and provided suggestions. A CVR above 0.80 indicates appropriate content validity for scale items (Davis, 1992; Delgado-Rico et al., 2012). Based on the experts’ opinions and the CVR, two items (10 and 49) were removed due to a low CVR, one item (28) due to similarity, and one item (26) was deemed unnecessary. This reduced the number of draft scale items from 61 to 57. The scale was a 5-point Likert scale, with response options ranging from 1 (strongly disagree) to 5 (strongly agree).

### 2.3. Phase 2: Validity and Reliability Study

#### 2.3.1. Sampling

To ensure maximum diversity, data were collected from three hospital groups: a Ministry of Health hospital, a university hospital and a private hospital, all of which were located in Istanbul, Turkey. Nurses working at the public university hospital were selected at random. Including participants with a wide variety of characteristics reduces sampling bias (DeVellis, 2017). The literature recommends that the sample size should be ten times the number of items on the scale. Since the draft scale consisted of 57 items, data were collected from at least 600 individuals (Watkins, 2018). Participants at the public university hospital were approached during working hours and the data collection tool was distributed by hand after consent was obtained. A total of 167 survey forms were collected from nurses on the same day; four declined to participate due to their workload. Nurses working in both private and public hospitals were contacted via Google Forms (Google LLC, Mountain View, CA, USA). Of those who responded to the consent question before the survey to indicate their acceptance of participation, 441 nurses were included in the study. A total of 608 datasets were obtained for the quantitative phase of the study. 

Following peer review, an additional data collection exercise was conducted to evaluate the robustness of the confirmatory findings. Between 27 August and 2 September 2026, an additional 45 nurses (44 women and 1 man, with a mean age of 34.6 ± 8.6 years) completed the final 36-item form. The inclusion criteria and informed-consent procedure were the same as before, with 20 nurses coming from the university hospital, 17 from Ministry of Health hospitals and 8 from private hospitals. The supplementary data collection wave was conducted under the same ethics approval as it involved the same target population, inclusion criteria, data collection procedure and informed consent process as the original study. Additional participants were not included in the exploratory factor analysis or item-selection process; the original confirmatory subsample (*n* = 304) remained the primary CFA sample. The additional data were only used for supplementary robustness analyses, which involved combining them with the original confirmatory subsample (*n* = 349), and for supplementary measurement-invariance analyses (combined *N* = 653).

#### 2.3.2. Construct Validity and Reliability

To avoid testing the factor structure on the sample from which it was derived, the original 608 questionnaires were randomly divided into two subsamples: an exploratory subsample of 304 and an independent confirmatory subsample of 304. This approach was taken in line with previous studies on the development of nursing scales (Çelebi Çakıroğlu & Baykal, 2021). The exploratory factor analysis, item-retention decisions and primary confirmatory analysis were based exclusively on the original sample. The 45 nurses recruited during the revision process only completed the final 36-item form, and their responses were used in supplementary robustness analyses (enlarged confirmatory sample, *n* = 349) and measurement-invariance analyses (combined *N* = 653). They did not contribute to the revision of the item composition or factor structure.

Exploratory factor analysis: Because the items were expected to reflect correlated latent attitudes rather than independent constructs, the common-factor model was used: principal axis factoring with promax rotation (κ = 4) (Watkins, 2018). Sampling adequacy was assessed using the Kaiser–Meyer–Olkin measure (≥0.70 for overall and item-level) and Bartlett’s test. The number of factors was determined using Horn’s (1965) parallel analysis (95th percentile of 300 random datasets), together with the interpretability of the rotated solution. Items were retained if their pattern coefficient on the primary factor was ≥0.40, if the difference from the largest secondary coefficient was ≥0.20, and if the item content was consistent with that of the other items in the same factor.

Confirmatory factor analysis: Because the items were five-point ordinal variables, several of which showed strong negative skewness and ceiling effects, the primary CFA was estimated using polychoric correlations with the diagonally weighted least squares (DWLS) estimator and mean- and variance-adjusted test statistics (WLSMV) in the original confirmatory subsample (*n* = 304). This is an appropriate estimator for ordinal indicators (Li, 2016). Maximum likelihood (ML) estimates were retained alongside WLSMV for comparability with the original submission and the wider scale-development literature. In response to the reviewer’s request for a fuller re-examination of the measurement model, competing models, residual diagnostics and theory-based residual covariances were evaluated in the enlarged robustness sample (*n* = 349). The fit of the final model was then compared directly with that obtained in the original confirmatory subsample. Residual covariances were admitted only between items belonging to the same factor that shared a specific content facet and had been presented consecutively in the questionnaire (Cole et al., 2007). Fit was evaluated with χ^2^(df), χ^2^/df, CFI, TLI, NFI, GFI, RMSEA with its 90% confidence interval, and SRMR; the indices were interpreted jointly rather than by treating conventional cutoffs as universal pass/fail rules (McNeish & Wolf, 2023).

Convergent and discriminant validity: Composite reliability (CR) and average variance extracted (AVE) were computed from the final model’s standardised loadings (Fornell & Larcker, 1981). Discriminant validity was assessed using the Fornell–Larcker criterion (where the square root of the AVE for each factor exceeds its correlations with the other factors) and the heterotrait–monotrait ratio of correlations (HTMT), with a conservative threshold of 0.85 (Henseler et al., 2015).

Measurement invariance: As a supplementary analysis requested by the reviewer, the configural, metric and scalar invariances of the final model were tested using multi-group confirmatory factor analysis (CFA) on the combined sample (*N* = 653), categorised by institution type (public: Ministry of Health and university hospitals vs. private hospitals) and sex. Invariance was accepted when the decrease in CFI did not exceed 0.010 and was accompanied by ΔRMSEA ≤ 0.015 and ΔSRMR ≤ 0.030 for metric invariance and ≤0.015 for scalar invariance (Chen, 2007). However, Δχ^2^ is oversensitive at this sample size and is therefore not reported. As the male subgroup was small (*n* = 70), sex-invariance findings were interpreted cautiously and as preliminary.

Reliability: The internal consistency of the final 36-item scale was primarily reported for the original scale-development sample (*N* = 608), using Cronbach’s α. To address possible redundancy in the items, supplementary evidence was included in the form of McDonald’s ω and mean inter-item correlations. The recommended range for constructs of moderate breadth is 0.15–0.50 (Clark & Watson, 2019). Inspection of highly correlated item pairs was also performed. Temporal stability was examined using a two-week test–retest procedure with 32 nurses.

### 2.4. Data Analysis

Qualitative data were analysed using Colaizzi’s (1978) inductive content analysis technique. Content validity indices were computed using the Davis (1992) technique. Descriptive statistics, item analysis, exploratory factor analysis and reliability coefficients were computed using IBM SPSS Statistics for Windows, Version 22.0 (IBM Corp., Armonk, NY, USA). ML confirmatory factor analyses and multi-group invariance models were computed using IBM SPSS Amos, Version 26.0 (IBM Corp., Armonk, NY, USA). Polychoric correlations, WLSMV confirmatory models, parallel analysis, CR, AVE and HTMT were computed using R, Version 4.6.1 (R Foundation for Statistical Computing, Vienna, Austria), with the lavaan (Rosseel, 2012) package, the psych (Revelle, 2024) package and the semTools (Jorgensen et al., 2022) package. Two data-entry errors in items not included in the final scale (one out-of-range value and one missing value) were replaced by the median value of the respective item. In this article, the authors used generative artificial intelligence (GenAI) only to make minor edits to the text (e.g., correcting grammar, spelling, punctuation and formatting).

## 3. Results

### 3.1. Descriptive Analyses

Of the nurses who participated in the original quantitative study, 57.9% were aged 30 or under (mean age = 32.17; standard deviation = 8.09). The majority were female (88.7%) and had a bachelor’s degree (75.8%). Participants worked in university hospitals (27.6%), Ministry of Health hospitals (41.3%) or private hospitals (31.1%). The 45 nurses in the second data-collection wave had a similar profile: 97.8% were women, with a mean age of 34.6 years, and 44.4% worked in a university hospital, 37.8% in a Ministry of Health hospital and 17.8% in a private hospital. In terms of professional experience, 63.8% of participants had worked for less than 10 years (mean = 9.51 years; SD = 8.24). Additionally, 64.3% had worked at their current institution for less than six years (M = 6.47 years; SD = 6.44) and 61.2% worked shifts.

### 3.2. Item Pool Construction

The scale items were developed through qualitative research. Four subcategories were identified and each item was allocated to the relevant sub-dimension. Fifteen experts assessed the 61 items on the draft scale using the Davis method. The experts were scholars specialising in Nursing Management, Psychiatric Nursing and Turkish Language, as well as individuals experienced in scale development. The CVI (Content Validity Index) was 0.90. Four items were eliminated due to similarity or because they were deemed unnecessary, leaving 57 items.

### 3.3. Validity and Reliability

The draft scale was used to collect data from participants. Data were collected from 608 nurses. Afterwards, validity and reliability tests were conducted on the data. To avoid introducing model-specific bias when performing both exploratory and confirmatory analyses on the same dataset, the database was randomly split into two. An EFA was performed on the first half of the dataset (*n* = 304) and a CFA on the model identified in the EFA was performed on the second half of the dataset (*n* = 304).

#### 3.3.1. Exploratory Factor Analysis

In the exploratory subsample (*n* = 304), the 57-item draft underwent factor analysis using principal axis factoring and promax rotation. The sampling adequacy was excellent (KMO = 0.93, with an item-level KMO of at least 0.70, and a Bartlett chi-squared value of 7967.42, with a *p*-value of less than 0.001). Twenty-one items were subsequently removed because they did not load on any factor (items 4, 6, 25, 31, 34, 36, 37, 38, 41, 47, 52, 54 and 55); loaded below the retention criterion (items 5, 18, 20, 26, 33, 39 and 40); or were the weakest of four items belonging to a factor whose content was inconsistent with that of the remaining three items (Table 1).

For the remaining 36 items, parallel analysis clearly supported three factors (with eigenvalues of 14.37, 3.37 and 2.46, compared to 95th-percentile random eigenvalues of 1.80, 1.69 and 1.61). A fourth factor was only marginally more significant than its random counterpart (with an eigenvalue of 1.67 versus 1.55), and a four-factor solution was not interpretable. The three-factor solution explained 52.3% of the common variance (38.6%, 8.1%, and 5.6%). All items had their largest pattern coefficient on the expected factor (range 0.41–0.89); the largest secondary coefficient was 0.35; and communalities ranged from 0.27 to 0.69 (Table 2). The factors were named F1: Perception and causes of violence (20 items); F2: Effects of violence (13 items); and F3: Response to violence (three items). F1 and F2 were moderately correlated (r = 0.61), whereas F3 correlated weakly with F1 (r = 0.16) and F2 (r = 0.19).

#### 3.3.2. Confirmatory Factor Analysis

**Figure 2:**
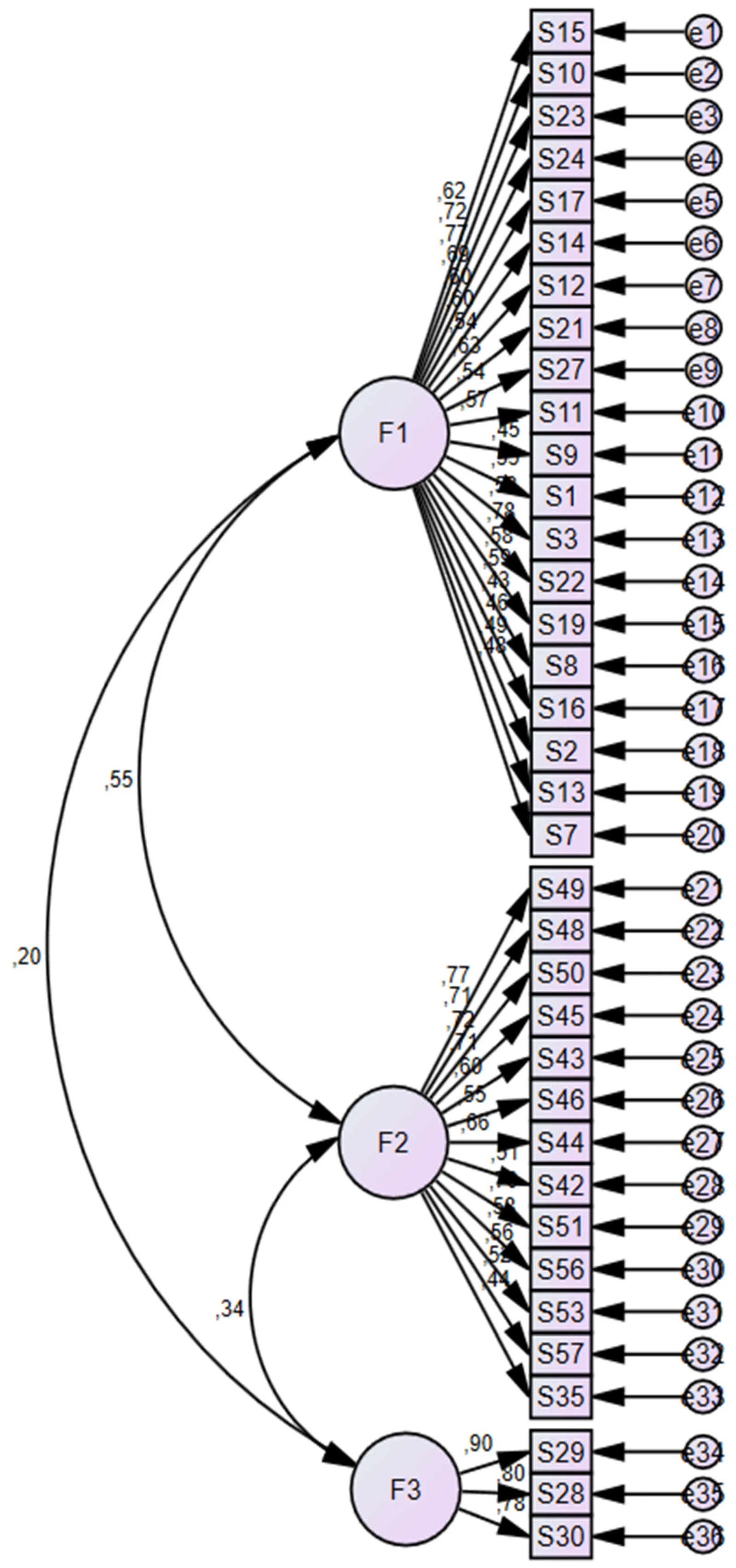
Initial first-order three-factor confirmatory factor analysis model (Model B; original confirmatory subsample, *n* = 304; standardized ML estimates).

**Figure 3:**
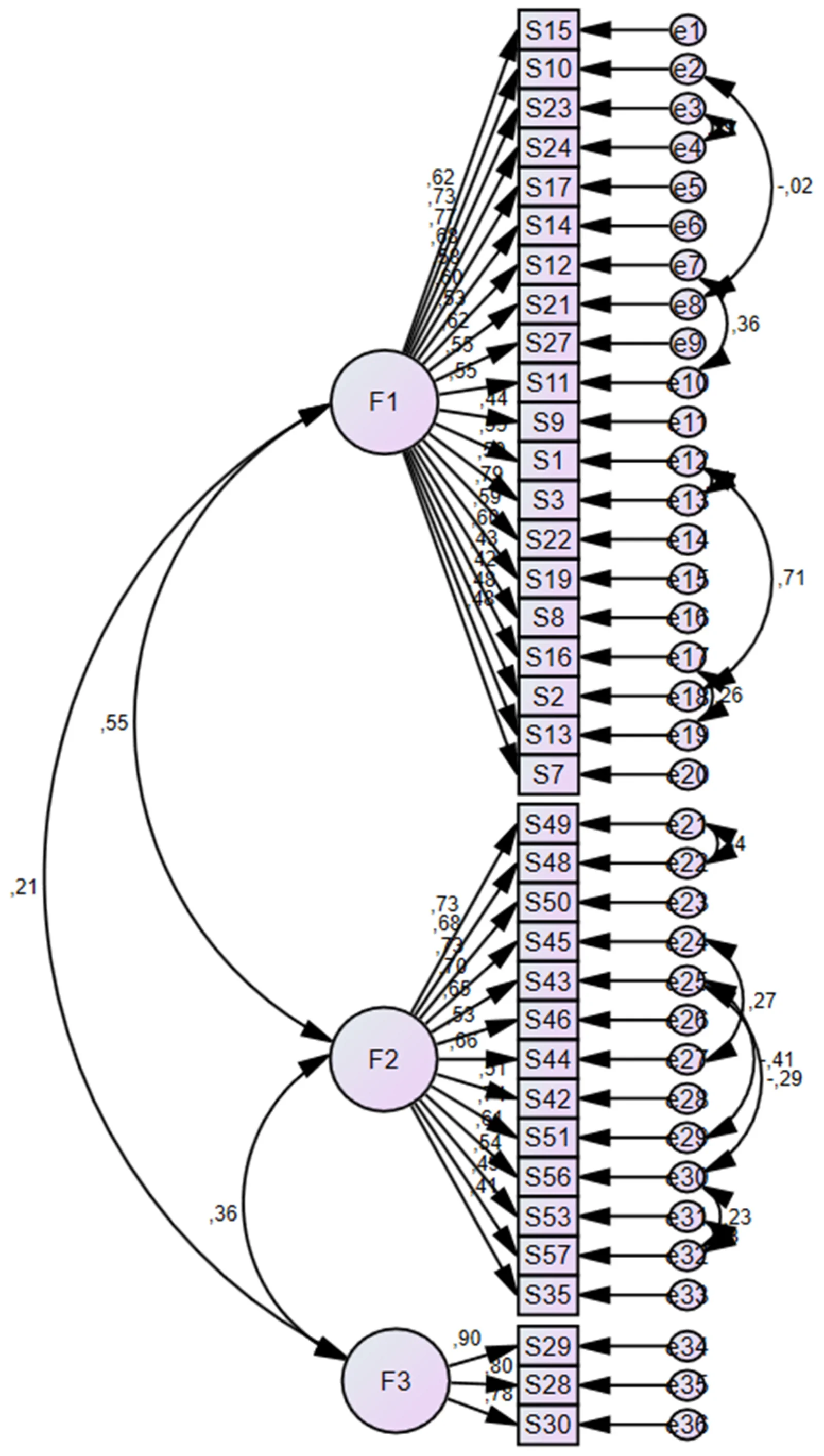
Final first-order three-factor model with six theory-based residual covariances (Model D; original confirmatory subsample, *n* = 304; standardized ML estimates).

The original confirmatory subsample (*n* = 304) was used to confirm the three-factor structure. Distributional diagnostics in the expanded robustness sample (*n* = 349) revealed significant non-normality and ceiling effects. All items were negatively skewed, except for the three response items. Notably, 87.4% of respondents selected ‘strongly agree’ for item 1, 77.7% for item 2, and 76.5% for item 16. Univariate skewness ranged from −4.54 to 0.47, kurtosis from −0.94 to 22.19, and Mardia’s normalised multivariate kurtosis was 46.9. Accordingly, WLSMV estimates were used primarily for the ordinal items. Figure 2 and Figure 3 show the initial and final three-factor models for the original confirmatory subsample. Table 3 reports the full competing-model re-estimation in the enlarged robustness sample, as well as the direct final-model comparison between *n* = 304 and *n* = 349.

In the expanded robustness sample (*n* = 349), the initial three-factor model (Model B) fit the data substantially better than the one-factor model, although the CFI remained below the conventional criterion (WLSMV CFI = 0.871, RMSEA = 0.072). Examining the standardised residuals and modification indices revealed a small number of theoretically interpretable within-factor item pairs, primarily among the initial normative statements and items with closely related content. The factor correlations were low to moderate (0.21–0.59), indicating that the remaining misfit was not due to factor redundancy. A four-factor model that separated perception from causes (Model C) produced only a modest improvement and did not yield a clearly distinct additional factor.

The final model (Model D) retained the three-factor structure and included six residual covariances based on theory between items with shared content within the same factor (Table 4). In the enlarged robustness sample, this model exhibited an acceptable overall fit under the weighted least squares mean and variance (WLSMV) estimator: χ^2^(585) = 1364.5, χ^2^/df = 2.33, CFI = 0.905, TLI = 0.898, root mean square error of approximation (RMSEA) = 0.062 [90% confidence interval (CI) 0.058–0.066], and standardised root mean square residual (SRMR) = 0.089. Notably, the same model produced highly comparable results in the original confirmatory subsample (*n* = 304): χ^2^(585) = 1297.5, χ^2^/df = 2.22, CFI = 0.900, TLI = 0.892, RMSEA = 0.063 [90% CI 0.059–0.068] and SRMR = 0.093. These highly similar results suggest that the conclusions drawn from the final model remained essentially unchanged following the inclusion of the additional cases. A more extensively modified model (Model E) produced only a small additional improvement in CFI (ΔCFI = 0.008), so it was not retained. All standardised factor loadings in the final model were statistically significant, and the original 36-item structure was retained.

#### 3.3.3. Convergent and Discriminant Validity

In the expanded robustness sample, the composite reliability score was high for all three factors (CR = 0.89–0.93). AVE was 0.74 for factor 3 (F3), approximately 0.50 for factor 2 (F2), and 0.40 for factor 1 (F1). Convergent validity was strongest for factor 3 (F3), while the AVE values for factors 1 (F1) and 2 (F2) were interpreted cautiously. Discriminant validity was supported as the square root of the AVE for each factor exceeded the corresponding inter-factor correlation, and all HTMT values were below 0.85 (Table 5). The original confirmatory subsample showed the same overall pattern.

#### 3.3.4. Measurement Invariance

In the combined sample (*N* = 653), changes in the fit indices met the pre-specified criteria for both metric and scalar invariance across institution type (ΔCFI = −0.005 and −0.009, respectively). Metric invariance was supported across sex (ΔCFI = −0.005), whereas full scalar invariance was only just supported (ΔCFI = −0.011). Allowing the intercept of item 25 to vary resulted in partial scalar invariance (ΔCFI = −0.009). As the male subgroup was small (*n* = 70), the sex-invariance findings should be considered preliminary (Table 6).

#### 3.3.5. Reliability

Internal consistency was primarily evaluated in the original scale-development sample (*N* = 608). Cronbach’s α was 0.943 for F1 (Perception and causes of violence), 0.930 for F2 (Effects of violence), 0.842 for F3 (Response to violence), and 0.950 for the total scale. Supplementary analyses conducted in response to the reviewer’s concern regarding possible item redundancy showed McDonald’s ω values of 0.89–0.93 across the three factors. Mean inter-item correlations were 0.40 for F1, 0.44 for F2, and 0.65 for F3. Only one item pair (items 1 and 2, r = 0.87) exceeded 0.75, suggesting that the high α of F1 was not attributable to widespread item redundancy, although local similarity between these two items was noted. Temporal stability was high, with a two-week test–retest correlation of r = 0.976 (*p* < 0.001) in 32 nurses.

Subscale scores are calculated as item means, ranging from 1 to 5. An increasing mean score in the first dimension (F1: Perception and causes of violence) indicates a stronger perception of violence and a greater recognition of perceived contributing factors. An increase in the mean score in the second dimension (F2: Effects of violence) suggests that nurses are being affected more severely by violence from patients and their relatives. An increase in the mean score in the third dimension (F3: Response to violence) indicates greater endorsement of the response patterns represented by the three retained items. As no empirical cut-off scores were established, subscale scores should be interpreted dimensionally rather than categorised as low or high. Due to the weak correlations between F3 and F1 and F2, it is recommended that subscale scores are interpreted rather than relying on a single total score.

## 4. Discussion

This study developed and validated the Nurses’ Attitudes Toward Violence by Patients and Patients’ Relatives Scale, a 36-item instrument with three dimensions grounded in nurses’ own accounts of violence in Turkish hospitals. Rather than restating the methods, we discuss here what the final structure means, why it differs from the four-domain framework that guided item writing, how the fit and validity evidence should be read, and what the instrument adds to the existing measures.

### 4.1. Meaning of the Three Dimensions

The first dimension, ‘Perception and causes of violence’, captures a set of evaluative-attributional beliefs: that violence is categorically unacceptable; that it has become normalised in healthcare; and that it is produced by identifiable antecedents located in patients and relatives (e.g., intoxication, negative previous experiences and misperceptions about neglect); in the nurse’s role (e.g., accessibility, enforcing rules and an inability to attend due to workload); and in the institution and society (e.g., inadequate security and sanctions, media image and emergency department crowding). This dimension therefore reflects the themes of ‘violence in healthcare’ and ‘violence against nurses’ from the qualitative phase, and is conceptually similar to the attribution-based factors of the Perception of Aggression Scale (POAS; Jansen et al., 1997). However, it is based on situations that nurses themselves described.

The second dimension, ‘Effects of violence’, spans emotional consequences (e.g., fear, anticipatory anxiety and feelings of helplessness), behavioural consequences (e.g., avoiding the violent patient, not entering the room alone and raising a code white) and professional identity consequences (e.g., feeling that one’s profession is worthless and that effort goes unrewarded, and loss of trust in people). The strongest indicators are anticipatory fear at work and fear of being followed. This mirrors the finding that the psychological consequences of workplace violence experienced by nurses are dominated by fear and hypervigilance rather than physical injury (Hou et al., 2022).

The third dimension, Response to violence, comprises three behaviours—remaining silent so that the incident does not escalate, ignoring negative remarks, and hesitating to react. Although the original factor label was retained, the three items predominantly represent passive or avoidant response tendencies. Higher scores therefore indicate greater endorsement of the response patterns captured by these items. The relatively weak correlations of F3 with F1 and F2 (0.21 and 0.32, respectively) suggest that responses to violence represent a comparatively distinct aspect of nurses’ attitudes toward violence.

### 4.2. Final Factor Structure

The item pool was initially organised under four content domains (perception, causes, response, and effects); however, the perception and causes domains did not emerge as clearly distinct factors. In the exploratory analysis, items from these two domains loaded on a single factor. Consistent with this finding, a forced four-factor model showed a correlation between the perception and causes factors ranging from 0.49 (ML) to 0.70 (WLSMV) in the confirmatory sample. Furthermore, only the three normative statements presented at the beginning of the questionnaire (items 1–3) substantially loaded onto the separate perception factor, while the remaining perception items had loadings below 0.45. When a fourth factor was extracted in the exploratory sample, it consisted primarily of the seven most strongly endorsed items (means 4.1–4.7) rather than mapping clearly onto a prespecified content domain; the three-factor solution was therefore retained on interpretability grounds. Conceptually, the idea of merging the two is plausible. Statements such as ‘violence in healthcare has become normalised’ or ‘nurses experience more violence than other healthcare workers’ are perceptions of violence and attributions of its causes. The nurses interviewed described what violence is and why it happens within the same narratives. We therefore interpret the first dimension as a single evaluative-attributional belief system. However, the three initial normative items share specific variance beyond this system. This is why their residual covariances were modelled. They are retained because they are anchored in the universal condemnation of violence expressed by every interviewee, and because removing them does not alter the fit of the model.

### 4.3. The Three-Item Response Dimension

The response domain initially included 14 items representing a range of strategies, such as seeking help, raising a code white, remaining calm, leaving the scene, pursuing legal rights, and remaining silent. These behaviours represent heterogeneous ways of responding to violence and did not converge on a single common factor. During factor analysis, several items were removed because of low or split loadings, while “raising a code white” loaded with the Effects of violence dimension. The three items retained in the Response to violence factor represent a narrower pattern characterised mainly by remaining silent, ignoring negative remarks, and hesitating to react.

In the original quantitative sample (*N* = 608), the mean scores were 2.49 (SD = 1.30) for remaining silent to prevent escalation, 2.57 (SD = 1.26) for ignoring negative remarks and 2.44 (SD = 1.28) for hesitating to react. As all three mean scores were below the midpoint (3) of the 1–5 response scale, the sample generally did not favour passive/avoidant response patterns.

Despite comprising only three items, this factor exhibited strong and consistent coefficients in both the exploratory and confirmatory analyses (EFA loadings = 0.72–0.79; WLSMV loadings = 0.83–0.89). The CR was 0.89 and the AVE was 0.74. While these findings support the internal coherence of the retained items, they also indicate that the factor captures a relatively narrow range of responses. Future studies may examine whether additional active-response items could broaden this dimension without compromising its factorial coherence. It is important to note that this factor does not indicate that nurses in the sample generally showed a ‘weak’ response to violence; rather, it reflects endorsement of the specific response patterns represented by the three retained items.

### 4.4. Interpreting Model Fit

The initial model misfit was concentrated in a limited number of same-factor item pairs with closely related content. After six theoretically justified residual covariances were added, the final WLSMV model showed overall acceptable fit in the original confirmatory subsample (CFI = 0.900, TLI = 0.892, RMSEA = 0.063, SRMR = 0.093). Highly comparable results were obtained in the enlarged robustness sample (CFI = 0.905, TLI = 0.898, RMSEA = 0.062, SRMR = 0.089), indicating that the overall interpretation of the model remained stable after inclusion of the additional cases. Although TLI remained slightly below 0.90, the fit indices were interpreted jointly rather than as evidence of excellent fit. WLSMV was treated as the primary estimator because of the ordinal and markedly non-normal item distributions, while ML results were retained for transparency and comparability. A sensitivity model with six additional residual covariances produced only a small improvement and was not retained, thereby limiting further data-driven modification.

### 4.5. Reliability, Validity, and Invariance

The high alpha of the first dimension in the original sample (α = 0.943) was examined directly rather than treated as evidence of superior reliability. In supplementary analyses, its mean inter-item correlation was 0.40, within the range recommended for constructs of moderate breadth (Clark & Watson, 2019), and only items 1 and 2 showed a consistently very high association. Their shared specific variance was modelled explicitly in the CFA. Thus, the findings do not indicate widespread redundancy across F1, although the local similarity of the opening normative items is acknowledged. As reported above, composite reliability was high, while the AVE of F1 remained below the conventional 0.50 benchmark. Rather than treating this as license to overlook the shortfall—composite reliability being a less conservative estimate that can mask a substantial share of error variance (Fornell & Larcker, 1981)—item loadings were all statistically significant, and inspection confirmed no single indicator was disproportionately relatively small number of male participants responsible for the low AVE. Discriminant validity was nonetheless supported by both the Fornell–Larcker and HTMT results, the latter considered more robust under marginal-AVE conditions (Henseler et al., 2015). Supplementary invariance analyses supported institution-type comparisons, whereas the sex-invariance findings remain preliminary given the small male subgroup (*n* = 70) and should be interpreted with caution pending replication in more balanced samples.

An additional interpretive consideration pertains to the construct itself. As highlighted in the Introduction, there is a potential divergence between formal definitions of violence and those based on lived experiences. The current scale was intentionally developed based on the latter; it includes items that reflect behaviors identified as violent by nurses during the qualitative phase, rather than adhering to a predetermined legal or clinical standard. Consequently, scores should be interpreted as representing the nurses’ own threshold for violence, which is influenced by cultural norms, previous exposure, and the type of behavior. It is important to note that physical acts are more readily classified than psychological or verbal ones. Therefore, cross-context comparisons should take this into account.

### 4.6. Contribution and Use of the Scale

Existing instruments primarily assess exposure to workplace violence or attitudes toward aggression in specific clinical settings, such as the POAS and HVAS-18. The present scale differs by being developed from nurses’ own accounts of violence by patients and relatives and by integrating evaluative-attributional beliefs, perceived effects, and responses to violence within a single instrument. Supplementary analyses also provided support for measurement invariance across institution type, although further validation in different samples is warranted.

The scale does not assess the frequency of violence exposure or the full range of coping strategies and should not be regarded as a diagnostic tool. It may be used to examine nurses’ attitudes toward violence across units or institutions, to evaluate changes following violence-prevention interventions, and as a measure in studies examining related outcomes such as reporting behaviour or turnover intention.

### 4.7. Limitations

This study has several limitations. Participants were recruited from hospitals in a single city, which may limit the generalisability of the findings. In the quantitative phase, part of the sample was reached through an online form, which may have introduced self-selection bias. The relatively small number of male participants (*n* = 70) also limited the strength of the sex-invariance analysis. As discussed in Section 4.5, the scale’s reliance on nurses’ self-perceived definition of violence, rather than a fixed legal or clinical criterion, should also be considered when interpreting and comparing scores across contexts. Model fit was stronger with the ordinal WLSMV estimator than with ML; in the original confirmatory subsample, CFI reached 0.900 whereas TLI remained slightly below 0.90. In addition, the final model included six theoretically justified residual covariances, which should be re-examined in future independent samples. Future studies should further examine criterion-related validity, measurement invariance, and the scale’s performance in larger and more diverse samples. Although the qualitative phase specifically included nurses with direct experience of violence, the quantitative phase did not classify or compare participants according to their prior exposure to violence. Future studies should examine whether scale scores differ according to the type and history of exposure.

## 5. Conclusions

The Nurses’ Attitudes Towards Violence by Patients and Patients’ Relatives Scale is a 36-item, three-dimensional instrument based on nurses’ own experiences and supported by exploratory and confirmatory evidence obtained from independent subsamples of the original study of 608 participants. The WLSMV confirmatory results were highly comparable in a supplementary robustness analysis that included an additional 45 nurses. The three dimensions—Perception and Causes of Violence, Effects of Violence, and Response to Violence—can be scored separately and represent distinct yet related aspects of nurses’ attitudes towards violence. The scale provides researchers and nurse managers with a multidimensional tool with which to examine how nurses perceive, are affected by, and respond to violence from patients and their relatives.

As the current sample was recruited exclusively from hospitals in Istanbul, it is recommended that further cross-cultural validation studies be conducted in different geographical regions, healthcare settings, cultural contexts and specialised clinical units, in order to confirm the scale’s psychometric properties and examine how attitudes towards violence may vary across organisational, cultural and individual contexts.

## Figures and Tables

**Table 1:** Items Removed in Exploratory Factor Analysis and Item Loadings.

Items	F1:	F2:	F3:
26. Poor management of tense situations increases the tendency for violence.	0.550		
18. Nurses’ harsh and rude speech sets the stage for the violence.	0.486		
20. Female nurses experience more violence.	0.481		
5. I perceive situations in which I am not respected as violent acts.	0.478		
39. After a violent incident, I exercise all my legal rights to the fullest.		0.508	
40. When I encounter violence, I try to manage the situation by controlling my anger.		0.483	
33. When subjected to violence, I seek help from a colleague.		0.483	
32. I attempt to resolve violent incidents without notifying security.			0.518

**Table 2:** Pattern coefficients, communalities, and corrected item–total correlations of the 36-item scale (principal axis factoring, promax rotation; exploratory subsample, *n* = 304).

Item	Content (Abridged)	F1	F2	F3	h^2^	r(it)
12 (S15)	People with negative hospital experiences show a tendency to violence.	0.844	−0.173	0.058	0.578	0.722
7 (S10)	Lack of respect for the nursing profession leads to violence.	0.821	−0.103	−0.012	0.578	0.730
18 (S23)	The negative perception created by the media leads to violence in health care.	0.742	0.031	−0.077	0.566	0.713
19 (S24)	Inadequate security measures in hospitals increase violence.	0.744	−0.064	0.043	0.511	0.683
14 (S17)	Alcohol and substance use increase the tendency to violence.	0.715	0.118	−0.114	0.611	0.751
11 (S14)	Failure to communicate an unexpected patient death appropriately leads to violence.	0.680	0.007	0.224	0.569	0.705
9 (S12)	Warning a patient/relative who breaks hospital rules leads to violence.	0.739	−0.272	0.107	0.400	0.567
8 (S11)	Nurses’ accessibility sets the stage for their exposure to violence.	0.659	−0.078	0.123	0.415	0.617
20 (S27)	Processing reactive patients faster leads others to become violent.	0.618	0.084	0.215	0.549	0.682
6 (S9)	Nurses experience more violence than other health workers.	0.649	0.064	−0.066	0.465	0.666
16 (S21)	Inadequate penalties for violence against health workers increase violence.	0.638	0.238	−0.226	0.633	0.711
15 (S19)	A nurse’s inability to attend to the patient because of heavy workload increases violence.	0.633	−0.083	0.304	0.488	0.616
1 (S1)	Violence is unacceptable under any circumstances.	0.630	0.286	−0.277	0.690	0.730
17 (S22)	Non-urgent patients presenting to emergency departments create an environment for violence.	0.610	0.117	0.110	0.512	0.671
3 (S3)	I perceive the violation of my personal boundaries as violence.	0.648	0.080	−0.152	0.477	0.664
13 (S16)	Violence is common when the patient is in pain.	0.663	−0.234	0.116	0.333	0.521
5 (S8)	I think violence in health care has been normalised.	0.605	0.126	0.014	0.479	0.670
2 (S2)	No living being should be subjected to violence.	0.619	0.231	−0.280	0.610	0.688
10 (S13)	The perception that health workers neglect their duties increases the tendency to violence.	0.605	0.114	−0.146	0.450	0.630
4 (S7)	I perceive speaking loudly as violence.	0.566	−0.113	0.078	0.272	0.498
31 (S49)	After experiencing violence, I come to work worrying about what I will go through today.	−0.141	0.887	0.056	0.673	0.767
30 (S48)	I am afraid that the violent patient/relative will follow me.	−0.077	0.856	0.056	0.677	0.774
32 (S50)	The violence I experience negatively affects my family and social life.	−0.016	0.795	0.013	0.620	0.766
28 (S45)	I feel uneasy when performing procedures on a violent patient.	0.071	0.713	0.048	0.591	0.735
26 (S43)	I take care not to go alone to the room of a violent patient.	0.015	0.715	0.022	0.531	0.684
27 (S44)	I try to spend less time with a violent patient/relative.	0.098	0.677	0.036	0.562	0.712
29 (S46)	After a violent incident, I have difficulty focusing on my work.	0.020	0.699	0.054	0.523	0.689
25 (S42)	I am afraid of violent incidents.	−0.124	0.671	0.204	0.448	0.586
33 (S51)	When I am subjected to violence, my trust in people decreases.	0.131	0.570	0.094	0.466	0.684
34 (S53)	Being subjected to violence makes me feel that my profession is worthless.	0.240	0.522	0.025	0.490	0.682
35 (S56)	After being subjected to violence, I feel helpless.	0.100	0.556	0.128	0.435	0.652
36 (S57)	When subjected to violence, I think my professional effort is not rewarded.	0.348	0.455	−0.130	0.503	0.642
24 (S35)	In a violent incident, I raise a “code white” alarm.	0.290	0.413	−0.028	0.396	0.563
22 (S29)	I ignore negative remarks; I do not care.	−0.054	0.139	0.787	0.659	0.765
21 (S28)	I remain silent so that the violent incident does not escalate.	−0.092	0.060	0.731	0.535	0.691
23 (S30)	I hesitate to react to violent situations.	−0.062	0.116	0.718	0.541	0.669

h^2^ = communality; r(it) = corrected item–total correlation with its own factor. Factor correlations: F1–F2 = 0.61, F1–F3 = 0.16, F2–F3 = 0.19. Sums of squared loadings after extraction: 13.90, 2.91, 2.02. KMO = 0.93; Bartlett χ^2^(630) = 7967.42, *p* < 0.001. The final scale numbers with the original draft numbers in parentheses.

**Table 3:** Fit statistics of the competing measurement models in the enlarged post-review robustness sample (*n* = 349).

(a) Maximum likelihood (AMOS 26)									
Model	χ^2^ (df)	χ^2^/df	CFI	TLI	NFI	GFI	RMSEA [90% CI]	SRMR	AIC
A. One factor	3767.8 (594.0)	6.34	0.486	0.454	0.446	0.539	0.124 [0.120, 0.128]	0.114	3911.8
B. Three factors, first order, no residual covariances (initial)	2543.8 (591.0)	4.30	0.684	0.663	0.626	0.678	0.097 [0.094, 0.101]	0.087	2693.8
C. Four factors (qualitative domains)	2205.0 (588.0)	3.75	0.738	0.719	0.676	0.719	0.089 [0.085, 0.093]	0.095	2361.0
D. Three factors + 6 residual covariances (final)	1919.8 (585.0)	3.28	0.784	0.767	0.718	0.753	0.081 [0.077, 0.085]	0.081	2081.8
E. Three factors + 12 residual covariances (sensitivity)	1775.0 (579.0)	3.07	0.806	0.789	0.739	0.769	0.077 [0.073, 0.081]	0.079	1949.0
(b) WLSMV on polychoric correlations (lavaan)									
Model	χ^2^ (df)	χ^2^/df		CFI			TLI	RMSEA [90% CI]	SRMR
A. One factor	3668.8 (594.0)	6.18		0.625			0.602	0.122 [0.118, 0.126]	0.147
B. Three factors, first order, no residual covariances (initial)	1652.6 (591.0)	2.80		0.871			0.862	0.072 [0.068, 0.076]	0.099
C. Four factors (qualitative domains)	1542.4 (588.0)	2.62		0.884			0.875	0.068 [0.064, 0.072]	0.094
D. Three factors + 6 residual covariances (final)	1364.5 (585.0)	2.33		0.905			0.898	0.062 [0.058, 0.066]	0.089
E. Three factors + 12 residual covariances (sensitivity)	1290.5 (579.0)	2.23		0.913			0.906	0.059 [0.055, 0.064]	0.086
(c) Robustness of the final model (Model D) in the original and enlarged confirmatory samples									
Index		*n* = 304 (original)						*n* = 349 (enlarged)	
WLSMV χ^2^ (df), χ^2^/df		1297.5 (585), 2.22						1364.5 (585), 2.33	
WLSMV CFI/TLI		0.900/0.892						0.905/0.898	
WLSMV RMSEA [90% CI]/SRMR		0.063 [0.059, 0.068]/0.093						0.062 [0.058, 0.066]/0.089	
ML χ^2^ (df), χ^2^/df		1879.6 (585), 3.21						1919.8 (585), 3.28	
ML CFI/TLI		0.769/0.752						0.784/0.767	
ML RMSEA [90% CI]/SRMR		0.085 [0.081, 0.090]/0.084						0.081 [0.077, 0.085]/0.081	

Note. All χ^2^ *p* < 0.001. WLSMV χ^2^ is the mean- and variance-adjusted statistic. A second-order model with three first-order factors is just-identified and has the same fit as Model B. Residual covariances in Model D: e1–e2, e1–e3, e2–e3, e8–e9, e18–e19, e34–e36; Model E adds e26–e27, e26–e28, e27–e28, e30–e31, e32–e33, e35–e36. Panel (c) shows that the final-model conclusions were essentially unchanged when the 45 post-review cases were added to the original confirmatory subsample.

**Table 4:** Standardized factor loadings of the final model (Model D) under WLSMV and ML, and residual covariances (enlarged post-review robustness sample, *n* = 349).

Factor	Item	λ WLSMV (SE)	z	λ ML	Unstd. ML (SE)		CR	R^2^ WLSMV	R^2^ ML
F1	12 (S15)	0.680 (0.032)	21.1	0.634	1.000 (—)		—	0.463	0.402
F1	7 (S10)	0.754 (0.029)	26.4	0.717	1.023 (0.090)		11.34	0.569	0.514
F1	18 (S23)	0.783 (0.028)	27.7	0.752	0.964 (0.082)		11.76	0.613	0.565
F1	19 (S24)	0.708 (0.038)	18.7	0.658	0.826 (0.078)		10.56	0.501	0.432
F1	14 (S17)	0.637 (0.038)	16.6	0.561	0.694 (0.075)		9.27	0.406	0.315
F1	11 (S14)	0.679 (0.033)	20.7	0.622	1.027 (0.102)		10.12	0.460	0.387
F1	9 (S12)	0.576 (0.036)	16.0	0.516	1.026 (0.119)		8.62	0.332	0.267
F1	8 (S11)	0.624 (0.036)	17.3	0.555	0.977 (0.106)		9.18	0.389	0.308
F1	20 (S27)	0.598 (0.037)	16.0	0.553	0.962 (0.105)		9.16	0.358	0.306
F1	6 (S9)	0.482 (0.049)	9.9	0.428	0.700 (0.096)		7.30	0.232	0.183
F1	16 (S21)	0.699 (0.039)	18.0	0.587	0.624 (0.065)		9.64	0.488	0.345
F1	15 (S19)	0.686 (0.034)	20.4	0.629	0.997 (0.098)		10.21	0.470	0.396
F1	1 (S1)	0.585 (0.066)	8.9	0.463	0.516 (0.066)		7.83	0.342	0.214
F1	17 (S22)	0.832 (0.023)	36.1	0.786	1.161 (0.095)		12.17	0.692	0.617
F1	3 (S3)	0.489 (0.043)	11.3	0.463	0.582 (0.074)		7.83	0.239	0.215
F1	13 (S16)	0.527 (0.038)	13.9	0.475	0.829 (0.103)		8.01	0.278	0.226
F1	5 (S8)	0.640 (0.035)	18.2	0.600	1.105 (0.113)		9.82	0.410	0.360
F1	2 (S2)	0.330 (0.055)	6.0	0.376	0.449 (0.069)		6.47	0.109	0.142
F1	10 (S13)	0.630 (0.033)	19.2	0.505	0.765 (0.090)		8.47	0.397	0.255
F1	4 (S7)	0.471 (0.043)	11.0	0.439	0.784 (0.105)		7.46	0.221	0.193
F2	31 (S49)	0.833 (0.022)	38.0	0.789	1.000 (—)		—	0.694	0.622
F2	30 (S48)	0.771 (0.025)	30.9	0.728	0.963 (0.068)		14.25	0.595	0.530
F2	32 (S50)	0.751 (0.024)	31.3	0.740	0.988 (0.068)		14.53	0.565	0.547
F2	28 (S45)	0.794 (0.024)	33.4	0.717	0.929 (0.066)		13.99	0.631	0.514
F2	26 (S43)	0.629 (0.035)	18.2	0.578	0.715 (0.066)		10.91	0.395	0.335
F2	27 (S44)	0.672 (0.031)	21.9	0.592	0.762 (0.068)		11.19	0.451	0.350
F2	29 (S46)	0.699 (0.037)	19.0	0.574	0.749 (0.069)		10.82	0.489	0.330
F2	25 (S42)	0.594 (0.034)	17.6	0.546	0.795 (0.078)		10.22	0.353	0.298
F2	33 (S51)	0.803 (0.023)	34.3	0.700	0.904 (0.067)		13.59	0.645	0.489
F2	34 (S53)	0.654 (0.041)	16.0	0.504	0.599 (0.064)		9.35	0.428	0.254
F2	35 (S56)	0.662 (0.034)	19.3	0.585	0.931 (0.084)		11.05	0.439	0.343
F2	36 (S57)	0.720 (0.039)	18.4	0.480	0.571 (0.064)		8.86	0.519	0.230
F2	24 (S35)	0.518 (0.051)	10.2	0.442	0.534 (0.066)		8.11	0.268	0.195
F3	22 (S29)	0.886 (0.022)	41.0	0.875	1.000 (—)		—	0.785	0.766
F3	21 (S28)	0.834 (0.024)	34.2	0.807	1.011 (0.064)		15.88	0.696	0.651
F3	23 (S30)	0.850 (0.026)	33.2	0.769	0.934 (0.061)		15.25	0.723	0.591
Residual covariances									
Pair		Shared content facet	WLSMV estimate (SE)		z	ML estimate (SE)	CR	ML standardized r	
e1 ↔ e2		Normative definition of violence	0.657 (0.051)		13.0	0.302 (0.027)	11.21	0.779	
e1 ↔ e3		Normative definition of violence	0.413 (0.058)		7.1	0.216 (0.025)	8.76	0.554	
e2 ↔ e3		Normative definition of violence	0.366 (0.057)		6.5	0.224 (0.027)	8.29	0.512	
e34 ↔ e36		Professional devaluation	0.276 (0.040)		6.9	0.379 (0.045)	8.39	0.530	
e8 ↔ e9		Nurse-role antecedents	0.220 (0.041)		5.4	0.279 (0.052)	5.35	0.315	
e18 ↔ e19		Institutional antecedents	0.188 (0.035)		5.4	0.093 (0.018)	5.08	0.326	

Note. Factor correlations (WLSMV/ML): F1–F2 = 0.59/0.52; F1–F3 = 0.21/0.21; F2–F3 = 0.32/0.32. SEs of WLSMV loadings are robust (sandwich) standard errors. CR = critical ratio. Marker items (unstandardized loading fixed to 1): items 12, 31, and 22.

**Table 5:** Composite reliability, average variance extracted, latent correlations, HTMT ratios, and Fornell–Larcker criterion (final model; enlarged post-review robustness sample, *n* = 349).

Factor	k	CR	AVE	√AVE	CR (ML)	AVE (ML)	r F1	r F2	r F3	HTMT F1	HTMT F2	HTMT F3
F1 Perception & causes	20	0.928	0.398	0.631	0.906	0.332	—	0.585	0.212	—	0.601	0.196
F2 Effects	13	0.927	0.498	0.706	0.889	0.387	0.585	—	0.324	0.601	—	0.308
F3 Response	3	0.893	0.735	0.857	0.858	0.670	0.212	0.324	—	0.196	0.308	—

Note. CR, AVE, r, and HTMT were computed from the WLSMV solution unless labelled ML. HTMT was based on polychoric correlations (Pearson-based HTMT: 0.58, 0.21, 0.33). The Fornell–Larcker criterion is met when √AVE exceeds the corresponding inter-factor correlations.

**Table 6:** Supplementary measurement invariance of the final model across institution type and sex (combined sample, *N* = 653; ML).

(a) Public (*n* = 456) vs. private hospitals (*n* = 197)								
Model	χ^2^ (df)	CFI	RMSEA	SRMR	Δχ^2^ (Δdf)	ΔCFI	ΔRMSEA	ΔSRMR
Configural	6352.5 (1170)	0.678	0.082	0.091	—	—	—	—
Metric	6471.5 (1203)	0.673	0.082	0.098	119.0 (33)	−0.005	−0.000	0.007
Scalar	6656.0 (1236)	0.663	0.082	0.098	184.5 (33)	−0.009	0.000	0.000
(b) Women (*n* = 583) vs. men (*n* = 70)								
Model	χ^2^ (df)	CFI	RMSEA	SRMR	Δχ^2^ (Δdf)	ΔCFI	ΔRMSEA	ΔSRMR
Configural	4961.0 (1170)	0.746	0.071	0.089	—	—	—	—
Metric	5069.1 (1203)	0.741	0.070	0.095	108.1 (33)	−0.005	−0.000	0.006
Scalar	5260.9 (1236)	0.731	0.071	0.096	191.7 (33)	−0.011	0.000	0.001
Partial scalar (intercept of item 25 free)	5231.8 (1235)	0.732	0.071	0.096	162.6 (32)	−0.009	0.000	0.000

Note. All Δχ^2^ *p* < 0.001. Invariance accepted when ΔCFI ≤ 0.010, ΔRMSEA ≤ 0.015, and ΔSRMR ≤ 0.030 (metric)/0.015 (scalar) (Chen, 2007).

## Data Availability

The data supporting the findings of this study are available from the corresponding author upon reasonable request. The data are not publicly available due to privacy considerations relating to the sensitive nature of the participant-level data on workplace violence (including qualitative interview data) and to protect the confidentiality of the participants.

## Abbreviations

The following abbreviations are used in this manuscript:

CVI	Content Validity Index
ILO	The International Labour Organization
ICN	International Council of Nurses
WHO	World Health Organization
PSI	Public Services International
CVR	content validity ratio
EFA	Exploratory Factor Analysis
CFA	Confirmatory Factor Analysis
x^2^/df	Chi-square goodness of fit index
RMSEA	Root Mean Square Error of Approximation
CFI	Comparative Fit Index
NFI	Normed Fit Index
SRMR	Standardized Root Mean Square Residual
TLI	Tucker–Lewis Index
GFI	Goodness of Fit Index
WLSMV	Weighted Least Squares Mean- and Variance-adjusted
ML	Maximum Likelihood
PAF	Principal Axis Factoring
CR	Composite Reliability
AVE	Average Variance Extracted
HTMT	Heterotrait–Monotrait ratio
MI	Modification Index
POAS	Perception of Aggression Scale
HVAS-18	Hospital Violence Attitude Scale-18
